# Current and Emerging Cell Culture Manufacturing Technologies for Influenza Vaccines

**DOI:** 10.1155/2015/504831

**Published:** 2015-03-01

**Authors:** Ernest Milián, Amine A. Kamen

**Affiliations:** Department of Bioengineeringt, McGill University, Macdonald Engineering Building, 817 Sherbrooke Street W, Montreal, QC, Canada H3A 0C3

## Abstract

Annually, influenza virus infects millions of people worldwide. Vaccination programs against seasonal influenza infections require the production of hundreds of million doses within a very short period of time. The influenza vaccine is currently produced using a technology developed in the 1940s that relies on replicating the virus in embryonated hens' eggs. The monovalent viral preparation is inactivated and purified before being formulated in trivalent or tetravalent influenza vaccines. The production process has depended on a continuous supply of eggs. In the case of pandemic outbreaks, this mode of production might be problematic because of a possible drastic reduction in the egg supply and the low flexibility of the manufacturing process resulting in a lack of supply of the required vaccine doses in a timely fashion. Novel production systems using mammalian or insect cell cultures have emerged to overcome the limitations of the egg-based production system. These industrially well-established production systems have been primarily selected for a faster and more flexible response to pandemic threats. Here, we review the most important cell culture manufacturing processes that have been developed in recent years for mass production of influenza vaccines.

## 1. Introduction

The influenza virus is responsible for a global epidemic every year that infects millions of people and causes serious illness and death worldwide. In the United States, infection by flu viruses results in a cumulative hospitalization rate of 35.5 per 100,000 people, mostly affecting the elderly (88.1 per 100,000 population) or very young people (46.7 per 100,000 population) with 107 pediatric influenza-associated deaths (http://www.cdc.gov/). Vaccination remains the primary and most effective strategy for the prevention and control of influenza.

Three genera of influenza viruses (A, B, and C) are susceptible to infecting humans. The influenza virus is a segmented, negative-stranded RNA genome of the Orthomyxoviridae family. The eight RNA segments of the influenza A and B genome encode for the viral proteins: PB1, PB2, PA, M1, M2, NS NA, HA, and NP [1]. Recently, novel additional influenza proteins such as PB1-F2 [2], PB1-N40 [3], PA-X [4], PA-N155, and PA-N182 have been described [5].

The influenza A virus is divided into subtypes based on the most common surface antigens: hemagglutinin (HA) and neuraminidase (NA). Seventeen different HA and nine NA glycoprotein subtypes have been identified to date [6]. Only one subtype has been defined for the influenza B virus; however, two antigenically different lineages, Yamagata and Victoria, have been identified [7].

Influenza virions constantly change the amino acid sequence of the HA and NA proteins to escape from the host's immunological control [8] by point mutations in the protein sequence (antigenic drift) or by exchanging the HA and NA viral RNA from a different influenza subtype that has infected the same host (antigenic shift) [9, 10]. The reassortment process generates a new virus encoding completely new antigenic proteins that can lead to a pandemic if the human population is immunologically naïve to the new virus.

Due to the high mutation rate of the influenza virus, vaccine manufacturers must reformulate their products every year to ensure a good match between the HA and NA present in the vaccine and the circulating strain [11]. Most common seasonal flu vaccines combine antigens derived from the three circulating influenza strains: two strains, H1 and H3, for influenza A and one strain for influenza B, as recommended by the WHO for each season in the Northern and Southern Hemispheres (http://www.who.int/influenza/en/). There are two types of influenza vaccines: the inactivated influenza vaccines and the live attenuated influenza vaccines (LAIV). The most common formulation of the seasonal influenza vaccine is the trivalent inactivated vaccine (TIV) with 15 *μ*g of each component. Among the inactivated influenza vaccines there are three types: whole virus, split virus, and subunit [12, 13].

To improve the efficacy of their vaccines, a number of vaccine manufacturers developed quadrivalent influenza vaccine formulations for seasonal vaccination by including both influenza B lineages. Recently, the Food and Drug Administration (FDA) has approved a quadrivalent formulation in which an additional strain of influenza B is added [14].

The majority of the current licensed influenza vaccines are made using embryonated hens' eggs (Tables 1 and 2) and a production system established in the 1940s. Fertilized hens' eggs are used as minifactories operated in parallel for influenza virus replication [15]. Both inactivated and LAIV vaccines share similar egg-based production process steps. The LAIV whole virus is recovered and further purified [16]. In 2003, the FDA approved the commercialization of FluMist (MedImmune), the first influenza trivalent vaccine with live attenuated virus [16–18]. However, it is important to note that LAIV have been in use for nearly 30 years in Russia [19, 20].

The egg-based production system has been used for more than 60 years, and it is still the most extensively used method to generate the 500 million vaccine doses (estimated from Partridge and Kieny [21]). The increased demand and the threat of a pandemic outbreak have accelerated the introduction of new manufacturing strategies for influenza vaccine production. In case of a pandemic, the egg-based production system might not be sufficient to meet the global demand due to egg availability. The mean estimated yield of the egg-based vaccine is one vaccine dose per one to two eggs. Considering the current manufacturing capacity estimated at 1420 million doses [21], it would be necessary to increase by a factor of 1.5 the current capacity to supply the world's population within 1 year. This surge capacity appears not feasible with the egg-based system [18]. Therefore, triggered by pandemic preparedness plans of different Western countries, several expression systems and manufacturing processes have been evaluated as alternatives to egg-based production methods. For example, the US Human Health Services, through the Biomedical Advanced Research and Development Agency (BARDA), actively supported the development of alternative manufacturing strategies for influenza vaccine. One favored option is to use cell culture for vaccine production. In contrast to egg-based production processes, cell-based production technology allows manufacturers to respond to market needs faster and in shorter production cycles and also allows a greater surge capacity, greater process control, and a more reliable and well-characterized product. Cell-based production allows manufacturers to supply higher quantities of the vaccines in a shorter amount of time [22–24]. Moreover, the virus obtained in cell cultures has a higher similarity with the circulating strains, in contrast with the virus produced in eggs, which might have antigenic modifications [25–27]. Another advantage of not using eggs for vaccine production is the avoidance of egg components that could induce allergic reactions [28]. Furthermore, cell culture can be used for the generation of viral seeds, thereby avoiding the long and cumbersome corresponding process in eggs [11].

On the other hand, the production of influenza vaccine using cell culture has some disadvantages. Cells must be free from adventitious virus, and virus yields might be low [11, 29], requiring more cell line screening and extensive process intensification.

This review is focused on the different types of cell culture manufactured influenza vaccines currently approved as well as new approaches toward designing influenza vaccine candidates produced using cell culture technologies.

## 2. Logistical Challenges and Critical Timelines for Influenza Vaccine Manufacturing

### 2.1. Surveillance and Generation of Viral Seed Stocks

Seasonal influenza vaccine content is based on a surveillance system for monitoring influenza virus circulation (http://who.int/influenza/gisrs_laboratory/en/) (http://www.influenzacentre.org/centre_GISRS.htm). WHO officials collaborate with national health agencies to identify circulating strains that had a dominant presence during the previous vaccination season and that are likely to cause the flu during the following winter in the Northern and Southern Hemispheres.

The National Influenza Centre performs virus isolation on certain samples obtained from patients to determine if the virus grows in culture. The virus isolation is done using mammalian cells, such as Madin Darby canine kidney (MDCK) cells instead of using embryonated eggs as it was carried out in the past [11]. Higher isolation rates have been obtained using MDCK cells rather than eggs, especially for H3N2 strain [30–32]. The virus is identified using reagents provided by WHO Collaborating Centers. Representative samples are sent for a more detailed study to one of the Collaborating Centers for influenza reference and research based in London (National Institute for Medical Research), Atlanta (Centers for Disease Control), Melbourne (WHO Collaborating Center for Reference and Research on Influenza), Tokyo (National Institute for Infectious Disease), Memphis (WHO Collaborating Centre for Studies on the Ecology of Influenza in Animals), and Beijing (WHO Collaborating Centre for Reference and Research on Influenza).

The seed strains are generated by genetic reassortment by three laboratories using the seasonal influenza H1N1 and H3N2 viruses provided by the WHO Collaborative Centers [33]. The selected seasonal strains are therefore combined with an H1N1/A/PR/8/34 strain that grows at high yields in embryonated eggs [34]. The traditional method for generating reassortant virus is based on the coinfection of the two strains in eggs. An alternative method for the production of reassortant viruses is the reverse genetics. Different approaches in reverse genetics have been developed but this is a cell culture-based technique that uses plasmid DNA alone with or without helper virus [35–37] to generate the reassortant viruses. Excellent reviews on reverse genetics have been recently published [38, 39]. The use of reverse genetics has the potential to streamline the process of generating reassortant viruses and become a better alternative to the cumbersome process of generating reassortant virus by egg coinfection [18]. Recently, reverse genetics has been used to produce the reassortant for H5N1 virus [40, 41].

The reassortant virus is then distributed among all of the manufacturers to start their production campaigns for seasonal influenza vaccines. Although most of the working material for surveillance is obtained from cell isolates, these isolated viruses cannot be used for seasonal influenza vaccine manufacturing since the seed precursor virus must be generated in eggs due to prevailing regulations [33, 42]. It has been reported that although an influenza viral strain is isolated from cell culture in the surveillance phase, it might be reisolated in eggs to obtain the viral seed that will be distributed to the manufacturers. However, if the manufacturer is using a cell culture technology the virus may be de novo readapted to grow in mammalian cells. This may add considerable additional delays in cell culture manufacturing process timelines [43]. With the buildup of additional safety data, it is expected that the viral seed manufacturing will be more streamlined and integrated into cell culture production of influenza vaccines.

### 2.2. Timetable in Conventional Vaccine Manufacturing

**Figure 1:**
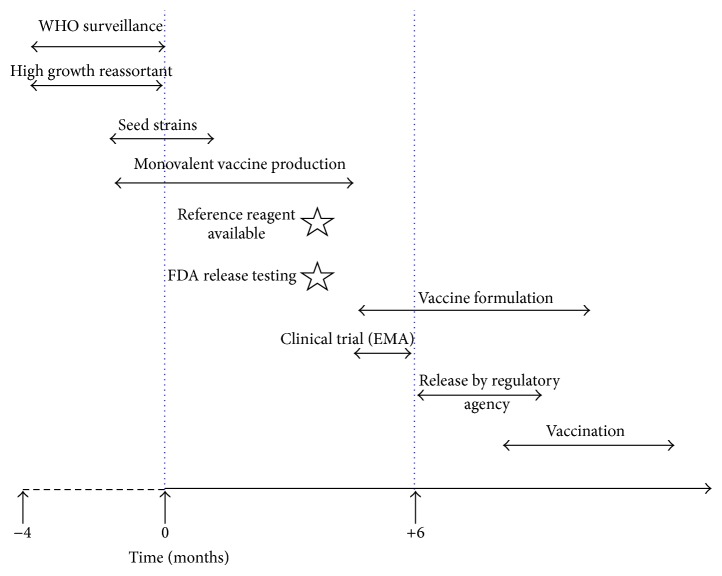
Timeline for seasonal influenza vaccine production in the Northern Hemisphere.

Seasonal influenza vaccine production is an enormous challenge for manufacturers because from the moment the WHO announces the seasonal strains in February for the Northern Hemisphere and in September for the Southern Hemisphere only a 6-month window is available for manufacturers to develop and supply the vaccines in July-August for the beginning of the vaccination campaign in September in the Northern Hemisphere and in March to start in April in the Southern Hemisphere (Figure 1).

Several factors may change the deadlines. One factor is the virus growth and a second factor is the fulfillment of all of the regulatory aspects [44]. These regulatory aspects include the control for viable influenza virus, the determination of HA content, and the presence of NA, testing the effective virus disruption, endotoxin, and the total protein content and performing sterility and stability tests on the final bulk vaccine [45].

In fact, to complete the trivalent or quadrivalent bulk vaccine formulation, it is vital to determine the quantity of HA antigen of each monovalent bulk using the single radial immune-diffusion (SRID) assay. SRID is the only validated potency assay, and its performance requires the availability of standard antigens and specific polyclonal antibodies. The preparation and distribution of the specific antibodies for SRID assays remains an additional cumbersome step that adds to the timelines for the formulation and clinical evaluation of the influenza vaccine. When the monovalent bulk has been quantified, the trivalent bulk can then be formulated. The trivalent bulk contains a minimum of 15 *μ*g HA of each strain per dose and has to be stable for 1 year.

Only four laboratories (Therapeutic Goods Administration (TGA), Australia; National Institute for Infectious Disease (NIID), Japan; National Institute for Biological Standards and Control (NIBSC), UK; Center for Biologics Evaluation and Research (CBER), USA) supply the reagents worldwide 3 months after the WHO has announced the candidate vaccine strains. However, obtaining the standardizing reagents can be a bottleneck in influenza vaccine production. Moreover, the European Medicines Agency (EMA) requires a previous clinical study of the new formulated vaccine before authorizing the market approval, forcing the manufacturers to release a formulated vaccine in June to complete the study [11, 17, 44].

## 3. Cell Culture-Based Vaccine Production

Building on major progress made in mammalian cell culture technology, influenza vaccine manufacturers have invested in cell culture for the mass production of influenza viral strains.

### 3.1. Influenza Vaccine Production Using Mammalian Cell Culture

Mammalian cell culture is a well-established technology for the production of therapeutic proteins or vaccines in the biopharmaceutical industry (HPV, polio, mAb) [46, 47]. Several mammalian cells have been evaluated for the production of influenza particles and, in particular, Madin Darby canine kidney cells (MDCK) [48, 49], human embryonic retinal cells (PER.C6) [50], monkey kidney cells (Vero) [51, 52], and human embryo kidney cells (HEK293) [47] have been successfully evaluated for industrial manufacturing of influenza vaccines.

**Figure 2:**
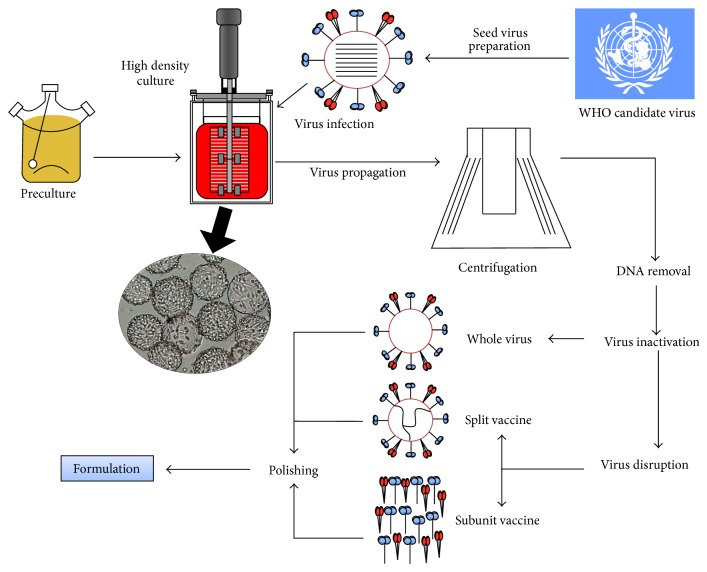
Schematic flowchart of the upstream and downstream production process of the cell-based inactivated influenza vaccine.

As early as 2001 Solvay Biologicals licensed in Netherlands Influvac a split virus vaccine produced in adherent MDCK cells. Production of Influvac was carried out in a serum-free medium using microcarriers (Cytodex 3) for cell attachment and intact virus was isolated using an affinity chromatography and then treated as an egg-based vaccine for inactivation [53]. Production of Influvac appears to be discontinued [54]. There are currently three FDA-licensed influenza vaccines produced in mammalian cells: Optaflu/Flucelvax (Novartis) and Preflucel and Celvapan (Baxter). A schematic representation of the influenza vaccine production and purification schemes is depicted in Figure 2.

### 3.2. Optaflu/Flucelvax

This vaccine was first approved in EU [54] and more recently by the FDA in 2012 [55]. It is a trivalent subunit vaccine composed of two influenza A (H1N1, H3N2) strains and one type B strain, produced in MDCK cells from egg-adapted influenza viral seeds. The MDCK 33016 cell line used grows in suspension in a serum-free and protein-free medium [53, 54, 56].

In this process, viral seeds recommended for the seasonal vaccination are expanded in MDCK 33016 cell culture. Progeny influenza viruses are recovered from the supernatant using sequential steps of centrifugation, filtration, chemical disruption, and chromatography to eliminate cell debris and separate the virus from the remaining component impurities. To limit the DNA content to less than 10 ng per dose Benzonase is added. The virus is inactivated by the addition of *β*-propiolactone (*β*-PL) and then disrupted by the addition of cetyltrimethylammonium bromide (CTAB) to solubilize the viral surface antigens HA and NA. The viral preparation is then ultracentrifuged [57]. Each of the three virus strains is produced and purified separately and then pooled to formulate the trivalent vaccine [54].

### 3.3. Preflucel and Celvapan

Preflucel is Baxter's seasonal influenza vaccine formulated with inactivated H1N1, H3N2, and influenza B produced in Vero cells licensed in EU in 2010 [58]. Vero cells grow on Cytodex 3 microcarrier. The cells are infected at a MOI of approximately 0.01 TCID_50 _mL^−1^ and the supernatant is clarified and treated with Benzonase for DNA degradation. Virus is inactivated by the addition of formalin. The downstream process continues with an ultracentrifugation for concentration and precipitation using protamine sulphate followed by a sucrose-gradient centrifugation, ultrafiltration, and sterile filtration for final formulation [51, 59–62]. Baxter developed the monovalent Celvapan for H5N1 or H1N1, which was approved for commercialization in Europe in 2009. Vero cells that were grown attached to Cytodex 1 or 3 microcarriers were used for the production of the vaccine [46]. Cells are infected by the H5N1 or H1N1 strain for viral production. The progeny virus is harvested and inactivated by the addition of formaldehyde and ultraviolet (UV) irradiation and then concentrated by sucrose gradient ultracentrifugation. The product is homogenized, and the sucrose is removed with the remaining impurities by an additional ultracentrifugation round. The final stage is a sterile filtration of the monovalent bulk [59].

### 3.4. Influenza Vaccine Production Using Insect Cell Culture

Insect cell and baculovirus expression vector (BEVS) systems have been extensively used for recombinant protein production. The BEVS method was successfully used to produce the human recombinant papillomavirus VLP cancer vaccine (Cervarix). BEVS and insect cells are being used to develop a number of VLP-based vaccines that are in preclinical studies (Ebola, Hantaan virus, hepatitis C virus, herpes simplex virus, and norovirus). Therefore, insect cells, with a well-accepted safety profile, are increasingly being considered for commercial vaccine production [63].

An alternative approach to the replication of the complete virus and the processing of virions for influenza vaccines is the exploitation of recombinant technology. This technology allows the expression of influenza virus antigens, such as monomeric or multiple HA, and also autoassembled fully folded proteins that can form virus-like particles.

Protein Sciences has licensed a trivalent vaccine of recombinant hemagglutinin (HA) produced in insect cell culture using a baculovirus expression system. FluBlok is the first recombinant HA influenza vaccine (trivalent) containing HA protein derived from the WHO recommended three influenza strains that are updated annually. The HA antigens included in FluBlok are full-length proteins containing the transmembrane domain and the HA1 and HA2 regions. The FluBlok formulation contains three times (45 *μ*g) the TIV quantity of HA for each of the three subtypes. It has demonstrated an excellent immunogenic capacity in young and older adults but not in children [64, 65].

**Figure 3:**
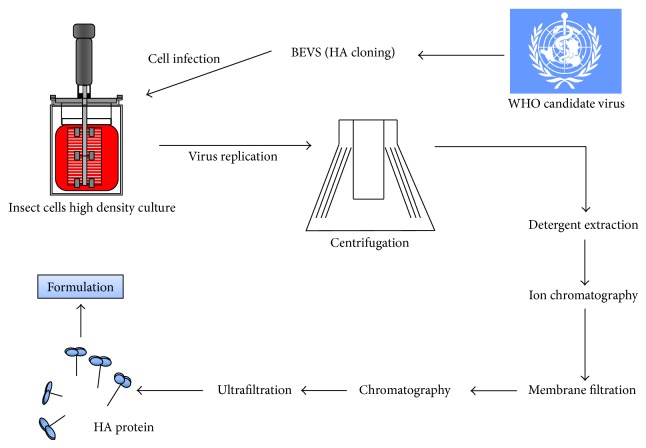
Schematic representation of the production process for the insect cell-based influenza vaccine FlubBlok.

Production of FluBlok (Figure 3) is carried out using the continuous insect cell line expressSF+, an insect cell line derived from Sf9 cells grown in serum-free medium. Each of the three recombinant HAs is expressed in the Sf9-derived cell line using a baculovirus vector [66].

When using the recombinant technology, there is no selection or adaptation of the influenza virus strains, allowing a good genetic match between the vaccine strains and the circulating influenza virus strains. Moreover, all of the production steps of FluBlok (cloning, expression, and manufacture) can be accomplished in a short period of time (less than 2 months) and do not require high-level biocontainment facilities when compared with pandemic strains that have to be manipulated under BSL3 conditions [42].

FluBlok production involves an initial cloning process of the HA gene into the baculovirus expression vector. The recombinant baculovirus generated is then used to infect insect cells in large-scale bioreactors. The infected cells are harvested using centrifugation, and the antigen is extracted from the cells using Triton X-100 and then clarified using depth filtration. The HA is purified by two column chromatography steps, an ion-exchange column followed by a hydrophobic interaction column, and two filtration steps, one filtration using a Q-membrane to remove residual DNA followed by an ultrafiltration step. The purified HAs are then blended and filled into single-dose vials [67, 68]. The overall production process starts at the reception of the virus sequences, and within 45 days the commercial production is initiated and the product is released after 75 days [68].

## 4. Emerging Technologies for Influenza Vaccine Production

The epitopes from the influenza virus structure, including the globular head of HA, the monomeric HA, or folded recombinant influenza proteins that generate a virus-like particle (M, HA, and NA), have been recombinantly expressed in different cell culture systems and other expression systems. The number of preclinical and early-stage development studies of recombinant candidate vaccines has increased [23, 69], indicating new trends and the adoption of modern technologies in the influenza vaccine industry.  Table 3 summarizes the current cell culture produced influenza candidate vaccines under development.

### 4.1. Cell-Based Produced LAIV

Researchers have also evaluated cell culture production of LAIV [70, 71] using either MDCK cells or Vero cells [70, 72]. Nobilon and MedImmune used adherent MDCK cells. MedImmune production process grows MDCK cells on Cytodex 3 microcarriers and serum-free medium to achieve a virus yield of 8.9 log_10_ FFU/mL, as measured by the fluorescent focus assay (FFA) [73], which is consistent with a high viral yield recovered from large-scale MDCK cell culture using roller bottles (1.0 · 10^9^ PFU·mL^−1^) [74]. Harvested viruses are filtered and clarified to remove cells. The clarified harvest is filtered (ultrafiltration or diafiltration) and Benzonase is added to digest the DNA. An affinity chromatography followed by a diafiltration is performed to remove residual MDCK DNA and proteins. The final step is a sterile filtration to recover the intact virus. Thus, a single use bioreactor with a working volume of 30 L would provide sufficient LAIV for the preparation of approximately 2.4 million doses of monovalent vaccine in a single run. A similar quantity of LAIV doses would have required 4000–8000 embryonated eggs [75]. Vivaldi Biosciences and AVIR Green Hills Biotech developed a deficient replication influenza virus by deleting the NS1 gene by reverse genetics and use Vero cells as cell factories for LAIV production [76–80].

### 4.2. Virus-Like Particles Influenza Vaccine

Virus-like particles (VLPs) are hollow structures composed of structural proteins that spontaneously assemble to yield particles with a similar morphology and antigenicity as the native virus. VLPs are genome-defective structures and are therefore unable to infect cells [67]. VLPs can be generated using methods similar to those used to generate complete viruses. Unlike split or subunit influenza vaccines, VLPs have the advantage of having a very similar surface antigen presentation as the native virus. Influenza VLPs form spontaneously when HA and M1 are coexpressed in a cell, with or without NA [23]. In the case of insect cells, the vehicle used to express the VLPs is a baculovirus coding for the genes of interest (Novavax).

The production of VLPs using baculovirus to infect insect cells is described by López-Macías et al. 2011 [81]. Briefly, VLPs are produced in Sf9 cells infected with recombinant insect baculovirus expressing HA, NA, and M1 genes. A recombinant baculovirus containing the three influenza genes was generated using a Bac-to-Bac expression system. Recombinant bacmid DNA is purified and transfected into Sf9 insect cells. Next, a single expressing HA, NA, and M1 recombinant baculovirus is identified, plaque-purified, and then amplified for use in the manufacture of the influenza A (H1N1) 2009 VLPs. The cGMP manufacture of recombinant VLPs is performed in a 100 L Wave Bioreactor with Sf9 cells infected with the recombinant baculovirus. H1N1 VLPs were harvested after 72 h using presterilized, disposable tangential flow filtration (TFF) assemblies for clarification, concentration, and diafiltration. The concentrated VLPs are then separated from host contaminants, baculoviruses, and nucleic acids using ion exchange chromatography, sucrose gradient ultracentrifugation, diafiltration in PBS, and a 0.22 *μ*m filtration step. The sterile purified H1N1 2009 VLPs are stored at 2–8°C and are stable when stored at this temperature for at least 1 year.

### 4.3. Vectored Influenza Vaccine

Another alternative influenza vaccine is the vectored influenza vaccine. This system uses replicating recombinant constructs based on a virus other than influenza to infect and immunize the subject. Different vector systems, such as adenovirus, poxvirus, parainfluenza virus, and alphavirus, have been used to express influenza HA and other influenza virus proteins in recombinant vaccine candidates for human seasonal as well as pandemic avian influenza viruses.

The main advantage of these vectors is their target sites that are similar to those of the influenza virus. Adenoviruses, for example, naturally target mucosal receptors. Adenovirus vectors have been used extensively as delivery systems to treat several infectious diseases (hepatitis B, rabies, anthrax, Ebola, SARS, and HIV-1) [82].

Defective adenovirus particles expressing HA delivered intranasally have proven potential to induce innate and adaptive immune response [23]. Several studies have been carried out using adenovirus as vectors. A phase 2 clinical study performed by Van Kampen et al. showed that both nasal and subcutaneous administration of an Ad5-HAPR8 to human volunteers elicits serum antibody titers but with high potency in the nasal administration [83]. HEK293 cells were used for the production of adenovirus. The vector was recovered by ultracentrifugation over a cesium chloride gradient followed by dialysis. Purified viruses were filter-sterilized, aliquoted, and stored at −80°C [84]. Another clinical trial (phase 1) using a recombinant adenovirus rAd expressing the HA of H5 avian influenza (rAd5-HA-TLR3) showed that oral administration of the vaccine had a positive effect on inducing immune responses to the antigen [85]. Moreover, the immune response of replicating vector-vaccine Ad4 expressing the HA from the avian influenza H5N1 was assessed in a phase 1 clinical trial. The vector was orally administered and induced a cellular immune response that increased with the administration of an H5N1 inactivated vaccine [86]. A different approach could involve the use of replication-deficient simian adenovirus expressing the influenza virus conserved proteins NP and M1. The vector vaccine induced a strong T-cell immune response against multiple influenza strains when tested in humans [87]. Mice models also showed high levels of humoral and cellular immune responses and were protected against virus replication after challenge with H5, H7, and H9 avian influenza virus [88]. Other studies in animal models have been performed and indicate sustained protective immunity against influenza virus [89, 90].

The small-scale production and propagation of adenovirus is sustained in an anchorage-dependent packaging cell line cultured in roller bottles, T-flasks, or cell factories [91]. The purification at this scale usually consists of a simple density gradient centrifugation by cesium chloride (CsCl), with yields of approximately 1 · 10^13^ viral particles (VPs) that are sufficient for clinical studies.

However, it is essential to develop large-scale production and purification processes to achieve the large quantities of viral vector (>10^15^ VPs) required for further clinical studies and commercialization.

Bioreactor systems can be used to produce adenovirus vectors using large-scale cell culture. Adenovirus vectors have been produced in large-scale bioreactor systems with microcarriers using human embryonic kidney (HEK) 293 cells, yielding 3-4 · 10^15^ VPs, or in suspension culture using PER.C6 and 293SF [82]. Wave bioreactors and stirred tank bioreactors are the main platforms used for the large-scale production of adenovirus vectors. Recoveries up to 5 · 10^15^ VPs have been achieved from a 500 L PER.C6 cell culture in a wave bioreactor expressing Ad5 [92]. More recently, a value of ~5 · 10^16^ VPs has been obtained for a replicating Ad5 (AdRG1.3) expressed in suspension culture of 293N3S cells in 500 L bioreactor [93].

Although the traditional CsCl purification system is valid for small-scale productions, it is not practical at large scale. Alternative purification methods are based on chromatography techniques. The purification process of adenovirus production consists of an initial cell lysis step by osmotic shock or microfluidization of the concentrated harvest. The cell lysate is treated with Benzonase for DNA removal and then centrifuged. The supernatant is filtered and goes through anion-exchange chromatography, with a recovery efficiency of 80%. The adenovirus is concentrated by ultrafiltration and then polished using a size-exclusion chromatography column, with the recovery of a 99% pure adenovirus [94]. The impact of the chromatographic steps on the adenovirus infectivity was demonstrated to be very low. This finding is a key factor in a pandemic scenario when large quantities of highly purified Ad-based vaccines would be required in a short period. The entire process from the identification of the vaccine virus to the vaccine formulation using the adenovirus vector-based vaccine technology is approximately 11 to 13 weeks [82].

## 5. Conclusions

Egg-based technology is still largely implemented among influenza vaccine manufacturers and will remain in use for many years, as it has demonstrated an outstanding robustness.

Meanwhile, several new influenza vaccine cell culture-based production methods have been granted commercial license within the last few years (Flucelvax/Optaflu, Celvapan, and Preflucel), whereas many influenza candidate vaccines using novel production technologies are gathering promising results in preclinical evaluation stages. FluBlok can be highlighted as one of the most relevant steps forward in the development of vaccines based on cell culture technology due to its more efficient production system. Recombinant vaccines have also risen as an alternative to the traditional influenza vaccine. Reverse genetics is a powerful tool to use in the influenza high growth reassortant development, simplifying the cumbersome conventional system. Cell-based LAIV proved their efficacy in phase 1 and phase 2 trials. The VLP influenza vaccines are also solid candidates to be considered as Novavax has concluded phase 2 clinical trials. Similarly, vectored vaccines, with special attention to adenovirus vaccines, showed great potential in clinical trials.

Despite advances, a pressing need for the traditional egg-based production remains and, for further development of cell culture-based vaccines, manufacturers need to render their vaccines as effective as possible and also deliver them as quickly as possible. These advances will be necessary to respond to a pandemic outbreak of influenza virus, which is predicted as a potential threat in the upcoming years.

The creation of a flexible and scalable system to supply influenza vaccine for the world's population while taking safety and cost-effectiveness into account remains one of the major challenges of the influenza vaccine industry and the national and international public health agencies.

## Figures and Tables

**Table 1:** FDA-licensed influenza vaccines for immunization and distribution in the USA for seasonal strains.

Product name	Commercial name	Manufacturer
Inactivated egg-based vaccines		
Influenza virus vaccine (trivalent)	Afluria	CSL Limited
Influenza virus vaccine (trivalent)	FluLaval	ID Biomedical Corporation of Quebec (a division of GlaxoSmithKline)
Influenza virus vaccine (trivalent)	Fluarix	GlaxoSmithKline Biologicals
Influenza virus vaccine (trivalent)	Fluvirin	Novartis Vaccines and Diagnostics Limited
Influenza virus vaccine (trivalent)	Agriflu	Novartis Vaccines and Diagnostics Limited
Influenza virus vaccine (trivalent)	Fluzone, Fluzone High-Dose, and Fluzone Intradermal	Sanofi Pasteur
Influenza virus vaccine (quadrivalent)	Fluarix Quadrivalent	GlaxoSmithKline Biologicals
Influenza virus vaccine (quadrivalent)	Fluzone Quadrivalent	Sanofi Pasteur
Influenza virus vaccine (quadrivalent)	FluLaval Quadrivalent	ID Biomedical Corporation of Quebec

Egg-based live attenuated influenza vaccines (LAIV)		
Influenza A (H1N1) 2009 monovalent intranasal	No trade name	MedImmune LLC
Influenza virus vaccine (trivalent) intranasal	FLuMist	MedImmune LLC

Inactivated egg-based vaccines for pandemics		
Influenza A (H1N1) 2009 monovalent vaccine	No trade name	ID Biomedical Corporation of Quebec
Influenza A (H1N1) 2009 monovalent vaccine	No trade name	CSL Limited
Influenza A (H1N1) 2009 monovalent vaccine	No trade name	Novartis Vaccines and Diagnostics Limited
Influenza A (H1N1) 2009 monovalent vaccine	No trade name	Sanofi Pasteur
Influenza virus vaccine (H5N1)	No trade name	Sanofi Pasteur

**Table 2:** Licensed influenza vaccines produced using cell culture technology.

Cell-based vaccines				
Product name	Commercial name	Manufacturer	Cell platform	Commercially available
Influenza virus vaccine (trivalent)	Flucelvax	Novartis Vaccines and Diagnostics Limited	MDCK	EU/FDA
Influenza virus vaccine (trivalent)	FluBlok	Protein Sciences Corporation	Insect cells	FDA
Influenza virus vaccine (trivalent)	Preflucel	Baxter	Vero	EU
Influenza virus vaccine (H5N1)	Celvapan	Baxter	Vero	EU
Influenza A (H1N1) 2009 monovalent vaccine	Celvapan	Baxter	Vero	EU

**Table 3:** List of influenza vaccines in clinical studies (http://www.clinicaltrials.gov/).

Technology	Company	Phase of development
Recombinant (VLP)		
VLP (HA, NA, and M) insect cell	Novavax	Clinical trial phase 2

Vectors		
Adenovirus	Paxvax	Clinical trial phase 2
Adenovirus	Vaxart	Clinical trial phase 1

Cell-based LAIV		
MDCK H5N1	MedImmune	Preclinical
dNS1-Vero	Vivaldi Biosciences	Phase 1
dNS1-Vero	AVIR Green Hills Biotech	Phase 2
